# Uncommon sarcomas of the uterine cervix: a review of selected entities

**DOI:** 10.1186/1746-1596-1-30

**Published:** 2006-09-18

**Authors:** Oluwole Fadare

**Affiliations:** 1 Department of Pathology, Wilford Hall Medical Center, Lackland AFB, San Antonio, TX, USA; 2 Department of Pathology, University of Texas Health Science Center at San Antonio, San Antonio, TX, USA

## Abstract

Sarcomas constitute less than 1% of all cervical malignancies. With over 150 reported cases, rhabdomyosarcomas represent the most commonly reported sarcoma at this location. In this report, a select group of the more uncommon sarcomas of the uterine cervix are reviewed, including all previously reported examples of leiomyosarcoma, liposarcoma, alveolar soft part sarcoma, Ewing sarcoma/primitive neuroectodermal tumor, undifferentiated endocervical sarcoma, and malignant peripheral nerve sheath tumor (MPNST). Emphasis is placed on any distinctive clinicopathologic features of these entities at this unusual location.

## Background

**Figure 1:**
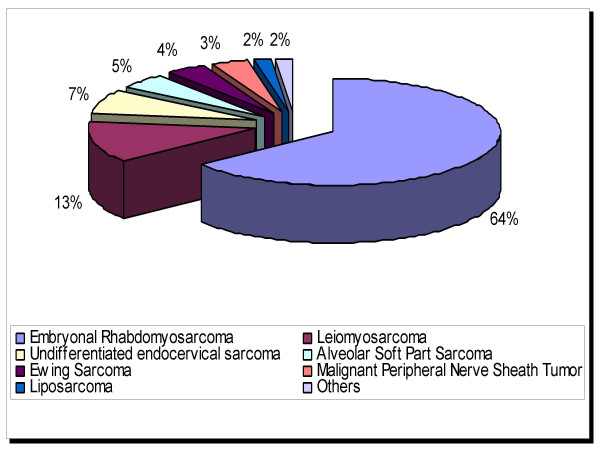
Proportional distribution (estimated) of all previously reported cases of cervical sarcoma.

In 2004, an estimated 10,520 new malignancies of the uterine cervix were diagnosed in the United States [1]. There are no current, systematically collected data on the precise percentage of these cases that were pure sarcomas. However, during a 5-year period in the United States (1973–1977), sarcomas constituted only 0.55% of all malignancies that were reported in the cervix [2]. Based on current data from individual institutions, and in the author's own experience, this proportion has remained largely unchanged. Wright et al [3] identified only 3 pure sarcomas out of 1583 cervical malignancies treated between 1986 and 2003 at a large tertiary center in the United States. [3]. The ratio of benign to malignant mesenchymal tumors at this anatomic location is approximately 1.9:1 [4]. Of the sarcomas, rhabdomyosarcomas, most commonly of the embryonal subtype, are the most frequently reported, with over 150 cases in the literature [4]. The proportional distribution of all previously reported cases of cervical sarcoma are summarized in figure 1. In this report, a select group of the more uncommon sarcomas of the uterine cervix are briefly reviewed, including all previously reported examples of leiomyosarcoma, liposarcoma, alveolar soft part sarcoma, Ewing sarcoma, undifferentiated endocervical sarcoma, and malignant peripheral nerve sheath tumor (MPNST). Since the basic pathologic features of most of these entities have been outlined in detail elsewhere in the soft tissue context [5], the emphasis is placed herein on any distinctive clinicopathologic features relating to the uterine cervix.

### 1. Leiomyosarcoma

Since cervical involvement by uterine corpus leiomyosarcomas are not uncommon, the diagnosis of a primary cervical leiomyosarcoma requires, at minimum, an attempt to exclude the possibility that the putative cervical tumor arose from the corporal isthmus (lower uterine segment). In the author's opinion, deference should be given to a corpus location in cases that are truly equivocal. However, reported examples of cervical leiomyosarcomas that developed in the cervical stump after supracervical hysterectomies provide sufficient evidence that leiomyosarcomas may indeed arise from this site [6,7] Approximately 30 cervical leiomyosarcomas have been reported [3,6-27]. They generally occur in the perimenopausal and postmenopausal population in their 4^th ^to 6^th ^decades of life. However, one distinctive case has been described in a pediatric patient [21]. Patients with cervical leiomyosarcomas most commonly present with abnormal vaginal bleeding and/or abdominopelvic pain. Macroscopically, the tumors are typically large (up to 12 cm in one series [9]), poorly circumscribed masses that either protrude from the cervical canal or thicken and expand it circumferentially [9]. Microscopically, they display a spectrum of morphologic subtypes similar to that seen in their corpus counterparts, including the myxoid variant [17], epithelioid variant [3,8,12,13,15], cases with an abundance of xanthomatous cells [16] and of course, conventional types. Furthermore, we have previously indicated [4] that we consider the "mesenchymal sarcoma", reported by Bader and Rundle [27] a probable example of leiomyosarcoma with osteoclast-like giant cells, another morphologic variation. The latter – osteoclast-like giant cells – are now well-recognized to occasionally occur in both endometrial stromal and smooth muscle tumors of the uterus [28,29]. An insufficient number of cervical leiomyosarcomas have been reported to devise the kind of elaborate morphologic criteria that are in routine use for uterine smooth muscle tumors. The current approach is to simply extrapolate diagnostic criteria from the corpus tumors and apply them to their cervical counterparts, incorporating various combinations of cytologic atypia, coagulative necrosis and mitotic activity to predict their malignant potential [4]. However, it is unclear if this is entirely appropriate in all cases [30].

Since therapeutic measures have been widely discordant amongst the reported cases, their true natural history and any variations in their malignant potential are not readily evaluable. In a 1983 review [31], the outcomes of 12 previously reported patients for whom follow-up information was available were as follows: Death of disease (n = 8), alive with recurrences within 2 years (n = 2), and alive after 5 years, disease status unstated (n = 2). In the largest series of 8 patients reported by Abell and Ramirez [9], 4 of the 6 patients that died did so of distant, hematogeneous metastases.

### 2. Malignant Peripheral Nerve Sheath Tumor

Malignant peripheral nerve sheath tumors (MPNST), also reported as "malignant schwannoma", "neurogenic sarcoma" and "neurofibrosarcoma", encompass any malignant tumor that shows differentiation "towards cells which are intrinsic to the peripheral nerve sheath" [32]. Eight cases of MPNST have been reported [33-38]. The 8 patients ranged in age from 25 to 73 years (mean 50 years). No predilection for patients with either of the neurofibromatosis syndromes was evident from a review of the reported cases. In contrast to leiomyosarcomas, the tumors in the reported cases of MPNST were generally smaller, presenting as polypoid masses measuring 3 to 4 cm. In general, the malignant nature of these tumors were evident upon their microscopic inspection, as they were comprised of an infiltrative and cellular proliferation of atypical, mitotically active spindle cells. The diagnostic dilemma is likely to arise from the failure to recognize their nerve sheath differentiation [4]. Subtle morphologic clues are present, however, which should cause one to include the appropriate markers in an immunohistochemical panel investigating an apparent cervical spindle cell sarcoma. The spindle cells may be arranged in herringbone, nodular or storiform fascicles [35-38]. In contrast to other sarcomas, the cells of MPNST tend to infiltrate but not destroy the native endocervical glands [36]. Occasionally, alternating hypocellular areas that may be myxoid, fibrous or edematous may be encountered [36]. Morphologic variations that have been reported include epithelioid areas in at least 2 cases [34,36], and pigmentation in one case [38]. Application of a judicious immunohistochemical panel is useful, especially in the distinction of cervical MPNST from leiomyosarcomas, as the former tumors are generally positive for S100 and vimentin and are negative for desmin, myoglobin and actin [36]. However, as is well-known, MPNST at all anatomic locations may display minimal to no immunoreactivity for S100. Meaningful follow-up information is only present in 4 (50%) of the 8 reported cases: 3 patients were alive without disease at 1–2 years follow-up [33,35,38], whereas the 4^th ^developed abdominal metastases approximately 1.7 years after initial treatment [36].

### 3. Ewing Sarcoma (Primitive Neuroectodermal Tumor)

Ewing sarcoma (primitive neuroectodermal tumor, EW/PNET), a primitive round cell sarcoma showing varying degrees of neuroectodermal differentiation [39], has rarely been reported in the uterine cervix. Nine previously reported cases of primary PNET of the cervix were retrieved in the current literature survey [40-47]. In contrast to osseous EW/PNET, in which the affected patients are generally less than 20 years old [39], the reported patients with cervical EW/PNET ranged in age from 21 to 51 years (mean 38 years). As with the aforementioned sarcomas, most of these patients present with abnormal vaginal bleeding. Preoperative radiographic analysis will generally show a well-circumscribed mass of approximately 5–7 cm, which caused its misinterpretation as a cervical leiomyoma in at least 2 cases [41,44]. Fadare et al [4] have previously summarized the outcomes in these 9 patients: Six of the 7 patients who presented with localized disease were alive without evidence of recurrent or metastatic disease at an average follow-up of 19 months (range 5–42 months). The seventh patient died 4.2 years after initial assessment with pulmonary metastases [46]. Initial management modalities for this group of 7 included various combinations of surgical resection, adjuvant or neoadjuvant chemotherapy [40,41,43,47] (with radiotherapy in 2 cases [45,46]). In two patients, the tumors were considered non-resectable [41,44]. For both patients, hysterectomies were preceded by neoadjuvant chemotherapy. One patient reportedly "achieved full remission" [41]. Follow-up information was not given in the other (44). Snijders-Keilholz et al [40] have recently advocated that the management approach to cervical EW/PNET be similar to their osseous counterparts: induction chemotherapy, surgery, and consolidation chemotherapy [40].

### 4. Alveolar Soft Part Sarcoma

Alveolar soft part sarcoma (ASPS), a tumor of uncertain differentiation, is comprised of a uniform population of large cells with eosinophilic to granular cytoplasm which are arranged in solid and/or alveolar nests [48]. As we have previously noted [4], in the gynecologic tract, ASPS apparently has a predilection for the cervix, as cervical cases comprise 11(38%) of the 29 cases that have been reported in this system [49-57]. The 11 patients with cervical ASPS ranged in age from 8 to 39 years (mean 29.9). In this respect, cervical ASPS are similar to their counterparts arising from the soft tissues, which most commonly occur in patients between 15 and 35 years [48]. However, some noteworthy differences exist between the tumors arising from these 2 locations (*vide infra*). In two of the 11 patients, their tumors were incidental discoveries during the examination of their uteri, which were resected for unrelated reasons. However, most patients (8 of the remaining 9) presented with abnormal vaginal bleeding or menstrual cramping [49-56]. Macroscopically ASPS are generally well-circumscribed (in contrast to their soft tissue counterparts), with tan to yellow cut surfaces and foci of hemorrhagic degeneration [50]. The 11 tumors ranged in size from 2 mm to 4 cm (mean 2.35 cm). [4]. Microscopically, cervical ASPS display the same distinctive morphologic features of their soft tissue counterparts. Notably, alveolar areas may be minimal and solid areas may predominate [50]. As shown in the series of Nielsen et al [50], mitotic activity is generally low and there should be no more than moderate nuclear atypia in cervical ASPS.

The propensity for soft tissue ASPS to show early metastases, especially to the thoracic and cranial cavities, is well-known [48]. No such propensity was identified based on the author's review of these 11 cases. However, it should also be noted that standard staging procedures were not performed for most of these cases. In one noteworthy case in which staging procedures were performed, a focus of metastatic tumor was present in an obturator node, even though no residual tumor was identified in the cervix following the initial biopsy diagnosis [57].

Overall, follow-up was available in all but 1 [49] case. In 8 (80%) of these 10 cases, there was no evidence of tumor metastases or recurrence with an average follow-up of 48 months (range 9–192). In the aforementioned case with an obturator lymph node metastasis [57], follow-up was largely unremarkable. Another patient experienced multiple recurrences of her tumor after initial treatments with cryotherapy prior to definitive diagnosis and treatment (hysterectomy) [54]. Most (82%) of the 11 patients were treated with a surgical intervention that included at least a hysterectomy. One patient received only radiotherapy after her initial biopsy [52] while another only received chemotherapy after an initial excision [50].

Although only a few cases of cervical ASPS have been reported, it is noteworthy that there have been no examples of pulmonary or brain metastases or death from disease. Cervical ASPS may have a better prognosis than soft tissue ASPS, although more cases are required to establish this possibility.

### 5. Undifferentiated Endocervical Sarcoma

Occasionally, sarcomas that show no specific line of differentiation at the light microscopic level are encountered in the cervix. Most of these tumors were reported prior to the routine use of immunohistochemistry, therefore it is probable that they are histogenetically heterogeneous. Abell and Ramirez [9], as well as Clement [58], have referred to these tumors as *endocervical stromal sarcoma*. In the current classification from the World Health Organization, however, they are designated *undifferentiated endocervical sarcoma *[59]. Seventeen examples of these cases have been reported [3,9,20,60,61] in patients ranging in age from 29 to 72 years (mean 51). As with most of the aforementioned sarcomas, the patients most commonly came to clinical attention due to abnormal vaginal bleeding. Their clinical appearances have been quite variable, ranging from protruding polypoid masses, ulcerated cervical masses or circumferential replacement of the cervix [9]. Indeed, in some patients, the tumors had such a non-descript appearance as to be initially misinterpreted as benign polyps [4]. The microscopic appearance of undifferentiated endocervical sarcoma is that of a moderate to high-grade, undifferentiated sarcoma. The constituent cells are stellate to spindle and are composed of moderately pleomorphic, hyperchromatic nuclei with minimal cytoplasm. Architecturally, the cells are configured in a sheet-like, storiform or fascicular pattern [9]. Mitotic figures are easily found, and finding greater than 10 mitotic figures per 10 high power fields in the most active areas of the tumor is typical [9,20,61,62]. Other findings in most cases include hemorrhage, necrosis, and stromal edema [9]. In one case [61], heterologous elements (cartilage) were identified.

The patient outcomes have been somewhat variable, which may be a reflection of the aforementioned probable histogenetic variability of these tumors. However, it may be stated that the outcomes for these patients have been generally unfavorable. Of the 14 patients with follow-up information, 4 patients were alive with no evidence of tumor recurrence or metastases at an average of 8 years after their diagnoses (range 2–18). Seven patients died of their disease within 2 years of their diagnoses. The three remaining patients showed evidence of either tumor recurrence or metastases: one patient developed 2 vaginal relapses both of which were excised, and she had no evidence of disease at 11 years of further follow-up [9]. Two patients showed radiographic evidence of pulmonary metastases 7–18 months after their initial evaluations. One of these patients [20] was lost to follow-up. In the other, the metastatic lesion was resected, and she was alive without evidence of disease after 7 years of additional follow-up.

### 6. Liposarcoma

Lipomatous tumors, whether benign or malignant, are extraordinarily rare in the uterine cervix [4]. The 1955 review of Bradfass et al [62] remains the definitive summary on the occurrence of lipomatous tumors in the uterus. These tumors may arise from the misplacement of an embryonic progenitor cell, metaplasia of mature mesenchymal tissues of other types, perivascular adipocytes or from traumatic displacement of adipocytes [62]. As one may anticipate, benign lipomas significantly outnumber liposarcomas at this location [4]. Indeed, to the author's knowledge, only four cases of pure liposarcoma of the cervix have been reported in the English literature [63-66]. The 4 patients had an average age of 54 years (range 45–62). Their tumors were generally large, with 3 of the 4 cases being ≥ 9 cm. Clinically and macroscopically, they formed protruberant polypoid masses with areas of gross hemorrhage. No histologic variant was predominant amongst the 4 cases: 2 were pleomorphic [63,65], 1 was round cell [66] and 1 was well-differentiated [64]. As expected, the 2 cases of pleomorphic liposarcoma [63,65] both recurred within a year after their primary resections. Follow up was information was unavailable in the 3^rd ^case and was unremarkable in the 4^th^.

## Summary and Conclusion

Cervical sarcomas constitute less than 1% of all cervical malignancies. With the exception of rhabdomyosarcomas, which have been well-characterized (4), the most commonly reported cervical sarcomas are reviewed herein. Each of these entities appear to have a distinctive clinicopathologic profile. Management options should be based on the outcomes of the previously reported examples of the entity in question. Indiscriminate direct extrapolations from their soft tissue counterparts may not be appropriate, at least for all entities.

## Competing interests

The author(s) declare that they have no competing interests.
